# Appendicitis Caused by a Giant Appendicolith

**DOI:** 10.7759/cureus.45780

**Published:** 2023-09-22

**Authors:** Christos Tepelidis, Athanasios Permekerlis, Panagiotis Fotiadis, Petros Kouridakis

**Affiliations:** 1 2nd Surgical Department, 424 General Military Hospital, Thessaloniki, GRC

**Keywords:** appendiceal perforation, acute peritonitis, rare case report, giant appendicolith, diagnosis of acute appendicitis

## Abstract

The coproliths of the appendix are accumulations of fecal remnants within its lumen. They are categorized based on their size into coproliths < 1cm, which are the most common, and giant coproliths, with a diameter > 2cm. It's important to note that the pathophysiology of acute appendicitis is characterized by the obstruction of the appendix lumen. This leads to distension due to the inability to expel secretions, ischemia, and ultimately rupture of its wall. This presentation discusses an interesting case of acute appendicitis caused by a giant coprolith. It also covers the clinical approach and information according to international literature. A 38-year-old man presented with sudden-onset right lower quadrant pain. Clinical examination revealed tenderness, a positive McBurney's point, elevated inflammation markers, and a radiopaque finding on an X-ray. A CT scan revealed a 2.5cm coprolith in the appendix. An exploratory laparoscopy revealed appendix wall rupture, followed by subumbilical incision appendicectomy and cleansing of purulent collection. The patient was discharged from the hospital on the fourth postoperative day without any complications, demonstrating a smooth recovery process. The presence of a coprolith predisposes the development of acute appendicitis. This condition is associated with a worse prognosis, as it increases the likelihood of perforation and the formation of intraperitoneal abscesses. This case underscores the clinical significance of giant coproliths as a potential etiology for acute appendicitis. Early recognition and timely surgical intervention are pivotal in achieving favorable patient outcomes.

## Introduction

Appendicitis, a condition marked by inflammation of the vermiform appendix, emerges as a significant medical concern with multifaceted clinical implications. It is characterized by the obstruction of the appendiceal lumen, often resulting from various factors encompassed within its etiology. The obstruction instigates an elevation in pressure within the appendiceal lumen due to the continuous secretion of fluids and mucus from the mucosa, coupled with the accumulation of these substances [1]. This intricate interplay between pressure elevation, bacterial proliferation, and vascular compromise underpins the progression from initial appendiceal obstruction to the complex cascade of events culminating in acute appendicitis.

In terms of epidemiology, appendicitis displays a distinctive pattern. It most commonly arises between the ages of five and 45, with an average age of onset around 28 years. Males exhibit a slightly higher predisposition to developing acute appendicitis compared to females. The lifetime incidence rates stand at 8.6% for men and 6.7% for women, respectively. Notably, this condition contributes to approximately 300,000 hospital visits annually in the United States alone [2].

Central to the etiology of appendicitis is the phenomenon of appendicoliths. These are masses formed by the aggregation of calcified deposits within the appendix, primarily composed of tightly packed stool and, at times, mineral deposits. Appendicoliths are notable in medical scenarios, being present in about 3% of the general population and found in roughly 10% of appendicitis cases [3]. Those >2 cm are described as giant and are uncommon [1]. Larger appendicoliths may be associated with more severe appendicitis They exhibit a higher prevalence in male patients under the age of 35, particularly those with a retrocecal appendix [4]. Despite being largely asymptomatic, appendicoliths emerge as a recognized cause of acute appendicitis and are linked to intermittent chronic abdominal pain. Furthermore, their presence increases the risk of appendix perforation and abscess formation, necessitating prompt clinical attention. Intriguingly, appendicoliths can also trigger colicky pain, warranting consideration of urolithiasis in differential diagnoses [3].

## Case presentation

**Figure 1:**
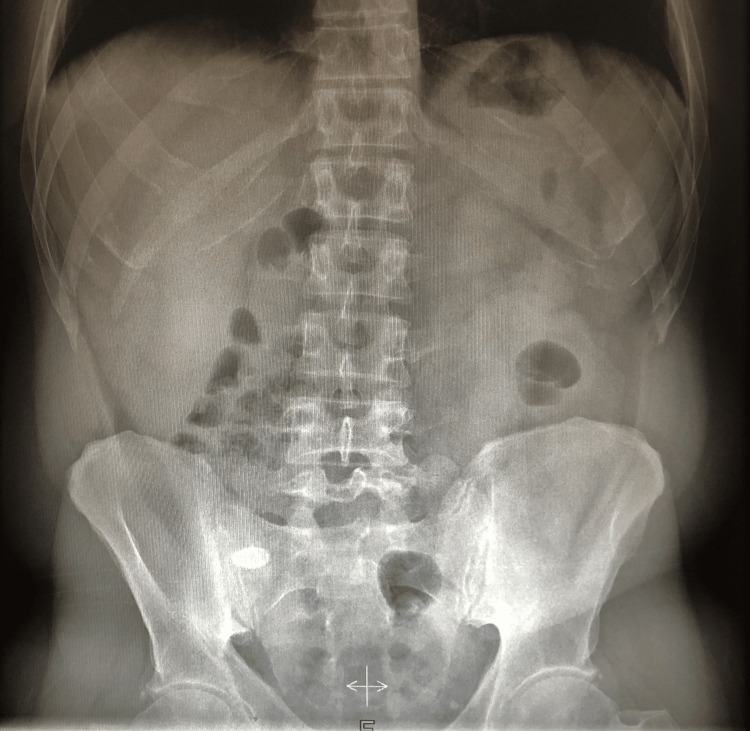
Abdominal X-ray showed a radiopaque finding.

A 38-year-old male, with no known past medical history and no history of previous surgeries, presented to the emergency department with a complaint of acute right lower quadrant abdominal pain that had been ongoing for the past 12 hours. The pain had a sudden onset and gradually worsened, without any associated symptoms such as loss of appetite, vomiting, changes in bowel habits, or fever. In clinical examination, the patient exhibited tenderness on deep palpation of the right lower quadrant of the abdomen, along with positive signs of peritoneal irritation (McBurney's point tenderness, Lanz point, Rovsing sign, Blumberg sign). Laboratory findings showed a hematocrit (Hct) of 38.4% (normal range: 38-52%), hemoglobin (Hb) level of 12.9 g/dl (normal range: 13.8-17.2 g/dl), white blood cell (WBC) count of 14.78 k/μL (normal range: 4.5-11 k/μL), and C-reactive protein (CRP) level of 11.18 mg/dl (normal value <0.5 mg/dl). Urinalysis was negative upon general examination. Subsequently, a plain abdominal X-ray was performed in an upright position, revealing a radiopaque finding in the right lower quadrant of the abdomen, precisely in the region where the patient reported pain (Figure 1).

**Figure 2:**
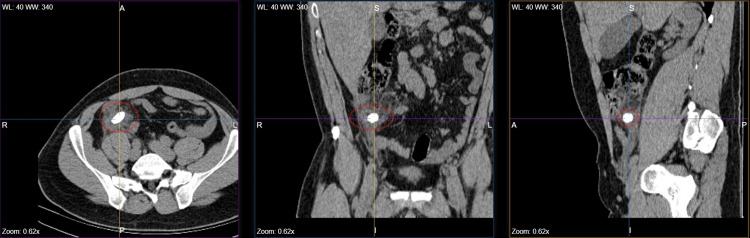
CT scan revealed a giant appendicolith, its exact size and location, and radiological signs of appendicitis.

Following the identification of the radiopaque finding and the clinical laboratory assessment, the decision was made for the patient to undergo a contrast-enhanced computed tomography (CECT) scan of the upper and lower abdomen (Figure 2). The results revealed the presence of a giant appendicolith in the lumen of the appendiceal orifice, measuring approximately 2.5 cm, along with radiological findings consistent with acute appendicitis, including thickening of the appendiceal wall and inflammation of the surrounding visceral fat. Subsequently, the patient was admitted to the Surgical Department, and was initiated on intravenous fluid administration, analgesics, and antibiotic treatment with piperacillin-tazobactam, due to its broad-spectrum coverage and clinical guidelines recommending its use for intra-abdominal infections.

After obtaining the patient's informed consent, they were prepared for surgery and taken to the operating room. We initiated the procedure with an exploratory laparoscopy, during which we encountered several challenging aspects. Anatomical landmarks, including the appendix's tip, the exact site of rupture, and its base, were not clearly identifiable during the laparoscopic procedure.

**Figure 3:**
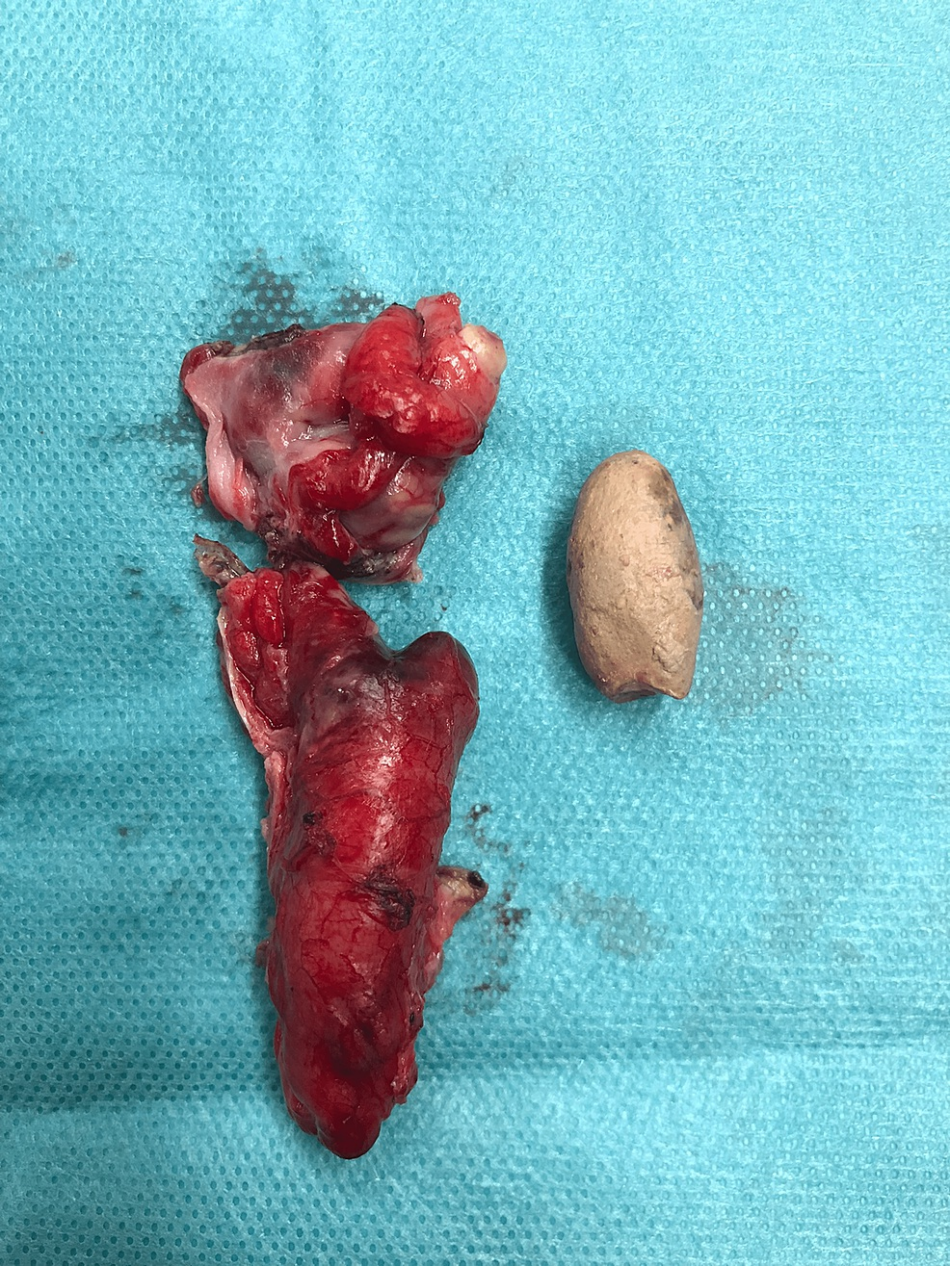
Surgical specimen of the appendix and the appendicolith. The appendix wall was only partially ruptured during the operation, and the surgical specimen was opened postoperatively to remove the appendicolith.

To optimize the patient's recovery and minimize potential complications, we made a subumbilical incision. The base of the appendix, where it connects to the cecum, appeared healthy and was readily visible with the open access. This observation significantly influenced our surgical approach. Subsequently, we performed the appendectomy, meticulously cleansing the area, and took steps to excise the affected portion of the appendix, including the appendix artery, while preserving the healthy base of the appendix. Figure 3 shows the surgical specimen of the appendix and the appendicolith. Following the successful appendectomy, we strategically placed an intraperitoneal drain to manage potential residual fluid and support the patient's recovery. The healthy base of the appendix played a critical role in guiding our surgical approach, ultimately aimed at improving the patient's outcome. This decision was made to minimize the need for mobilizing other structures, thereby reducing surgical complexity

The patient responded well to treatment, with resolution of symptoms, normalization of laboratory parameters, and clinical improvement. On the fourth postoperative day, due to the low quantity and good quality of drainage, the abdominal drain was removed. The patient's condition remained stable, and he was discharged from the hospital on the fourth postoperative day without any complications.

## Discussion

Clinical significance of appendicoliths in acute appendicitis

Acute appendicitis, a common surgical emergency affecting approximately 7% of the general population, is often triggered by the obstruction of the appendix lumen. Factors contributing to lumen obstruction include lymphoid hyperplasia, fecalith, stricture, and appendicolith. Appendicoliths, composed of firm stool and mineral deposits, represent a subset of obstructions and have been implicated in the pathogenesis of acute appendicitis [3].

Role of appendicoliths in appendicitis severity

Appendicoliths are known to influence the clinical course of appendicitis. The location and size of appendicoliths appear to be significant determinants of disease severity. Studies suggest that appendicoliths are more likely to be associated with complicated cases of appendicitis, including gangrenous appendicitis and perforation [5]. Notably, appendicoliths positioned at the root of the appendix have been linked to higher rates of perforation, possibly due to their propensity to exacerbate obstruction and subsequent inflammation [6].

Clinical presentations and diagnostic challenges

Appendicoliths may not always manifest as acute appendicitis. Asymptomatic cases are common and can be incidentally detected through imaging, such as CT scans, in otherwise normal appendices. However, symptomatic presentations of appendicoliths can mimic urolithiasis, posing diagnostic challenges. Abdominal pain, leukocytosis, and hematuria are shared symptoms, making it important to distinguish between these conditions for accurate management [3].

Radiological evaluation and management

Abdominal X-rays, ultrasonography (USG), and CT scans play pivotal roles in diagnosing appendicoliths and related conditions. In a retrospective review by Lowe et al., an appendicolith detected on CT had a sensitivity of 65%, specificity of 86%, and positive predictive value of 74% for the diagnosis of appendicitis [7]. These imaging modalities aid in detecting sufficiently calcified appendicoliths and differentiating them from other pathologies. However, it's crucial to highlight that in the published literature, graded-compression USG has shown an extremely variable diagnostic accuracy in the diagnosis of acute appendicitis, with reported sensitivity ranging from 44% to 100% and specificity ranging from 47% to 99% [8]. This variability underscores the need for a cautious approach, considering the potential limitations of USG, and suggests that, when in doubt, complementing USG findings with other imaging techniques, such as CT scans, can provide a more comprehensive and accurate assessment of acute appendicitis and appendicolith-related conditions. Such a multifaceted diagnostic strategy is especially critical given the high possibility of complications associated with appendicoliths, reinforcing the significance of meticulous medical history, the surgical approach, and the utilization of CT in achieving optimal patient outcomes.

Variability in appendicolith characteristics

Appendicoliths' prevalence and clinical implications exhibit significant variability. They are found in about 10% of acute appendicitis cases and are more frequently associated with perforation and abscess formation. The case of a giant appendicolith, exceeding 2 cm in size, is an unusual occurrence but can have pronounced effects on the severity of appendicitis [1,6].

Conservative management of asymptomatic giant appendicoliths

Giant appendicoliths, exceeding 2 cm, are rare findings in acute appendicitis. Typically, they are associated with symptomatic presentations and are managed through surgical removal. However, the report from Scroggie et al. presents a unique case where an asymptomatic patient with a giant appendicolith opted for conservative management due to medical history and patient preferences [1]. This case highlights the need for individualized decisions, taking into account the patient's condition, risk factors, and long-term considerations.

Future implications and research

The interplay between appendicoliths, disease severity, and treatment outcomes warrants further investigation. While appendicoliths are recognized as potential exacerbating factors, a comprehensive understanding of their mechanisms and effects is essential. Future research could explore the predictive value of appendicolith characteristics in determining the risk of complications, aiding clinicians in making informed decisions.

## Conclusions

Physicians should be mindful of the possibility of appendicoliths and utilize all available diagnostic tools for a swift and accurate diagnosis, enabling a tailored treatment plan. Appendicolith-related acute appendicitis carries the potential for serious complications, including perforation and peritonitis. Therefore, the surgical approach should remain individualized, with a focus on timely intervention to mitigate these risks. CT imaging remains pivotal in diagnosing appendicolith-related acute appendicitis, offering valuable insights into the condition's severity and guiding appropriate management strategies. Embracing a patient-centric approach, which includes personalized surgery and prudent CT utilization, stands as the cornerstone for optimizing outcomes in this context. As our understanding of appendicoliths and their implications continues to evolve, it reinforces the importance of a tailored, patient-centered approach in the management of appendicitis.
